# Supplementary material for: Rational Design of a Pan-Coronavirus Vaccine Based on Conserved CTL Epitopes
Source: Viruses. 2021 Feb 21;13(2):333. doi: 10.3390/v13020333 (PMC7926959; doi:10.3390/v13020333)
Supplement: Supplementary file 1 [file viruses-13-00333-s001.zip › supplementary DATA/Supplementary Data 1-Distribution of HLA allele frequency in different countries.docx]

**HLA-A*02:01**

| Region | Number of people typed (sample size) | Allele count | Allele frequency |
| --- | --- | --- | --- |
| Europe | 105521 | 28212 | 26.74% |
| North America | 2882614 | 580501 | 20.14% |
| South and Central America | 3287 | 624 | 18.98% |
| Australia | 503 | 83 | 16.50% |
| North Africa | 896 | 138 | 15.40% |
| South-East Asia | 23188 | 3289 | 14.18% |
| North-East Asia | 26405 | 3468 | 13.13% |
| Sub-Saharan Africa | 2385 | 285 | 11.95% |
| Western Asia | 196013 | 21523 | 10.98% |
| Oceania | 1135 | 72 | 6.34% |
| South Asia | 4786 | 257 | 5.37% |
| Worldwide Total | 3246733 | 638452 | 19.66% |

**HLA-A*11:01**

| Region | Number of people typed (sample size) | Allele count | Allele frequency |
| --- | --- | --- | --- |
| South-East Asia | 23188 | 6140 | 26.48% |
| Oceania | 1135 | 246 | 21.67% |
| South Asia | 4786 | 676 | 14.12% |
| Australia | 503 | 60 | 11.93% |
| North-East Asia | 26405 | 2425 | 9.18% |
| North America | 2882614 | 202107 | 7.01% |
| Europe | 105521 | 6426 | 6.09% |
| Western Asia | 196013 | 10130 | 5.17% |
| South and Central America | 3287 | 106 | 3.22% |
| North Africa | 896 | 24 | 2.68% |
| Sub-Saharan Africa | 2385 | 37 | 1.55% |
| Worldwide Total | 3246733 | 228379 | 7.03% |

**HLA-A*24:02**

| Region | Number of people typed (sample size) | Allele count | Allele frequency |
| --- | --- | --- | --- |
| North-East Asia | 26405 | 8626 | 32.67% |
| Oceania | 1135 | 344 | 30.31% |
| Australia | 503 | 113 | 22.47% |
| South and Central America | 3287 | 628 | 19.11% |
| South-East Asia | 23188 | 3920 | 16.91% |
| South Asia | 4786 | 724 | 15.13% |
| Europe | 105521 | 10464 | 9.92% |
| North America | 2882614 | 279153 | 9.68% |
| Western Asia | 196013 | 11200 | 5.71% |
| North Africa | 896 | 48 | 5.36% |
| Sub-Saharan Africa | 2385 | 57 | 2.39% |
| Worldwide Total | 3246733 | 315275 | 9.71% |

**HLA-A*33:03**

| Region | Number of people typed (sample size) | Allele count | Allele frequency |
| --- | --- | --- | --- |
| South Asia | 4786 | 503 | 10.51% |
| South-East Asia | 23188 | 2137 | 9.22% |
| North-East Asia | 26405 | 2281 | 8.64% |
| Oceania | 1135 | 74 | 6.52% |
| North America | 2882614 | 85990 | 2.98% |
| North Africa | 896 | 25 | 2.79% |
| Sub-Saharan Africa | 2385 | 61 | 2.56% |
| South and Central America | 3287 | 31 | 0.94% |
| Europe | 105521 | 632 | 0.60% |
| Australia | 503 | 1 | 0.20% |
| Western Asia | 196013 | 62 | 0.03% |
| Worldwide Total | 3246733 | 91798 | 2.83% |

**HLA-A*30:01**

| Region | Number of people typed (sample size) | Allele count | Allele frequency |
| --- | --- | --- | --- |
| North Africa | 896 | 56 | 6.25% |
| Sub-Saharan Africa | 2385 | 136 | 5.70% |
| South-East Asia | 23188 | 708 | 3.05% |
| Western Asia | 196013 | 5861 | 2.99% |
| North America | 2882614 | 72642 | 2.52% |
| South and Central America | 3287 | 68 | 2.07% |
| Europe | 105521 | 1792 | 1.70% |
| South Asia | 4786 | 77 | 1.61% |
| North-East Asia | 26405 | 206 | 0.78% |
| Oceania | 1135 | 6 | 0.53% |
| Australia | 503 | 2 | 0.40% |
| Worldwide Total | 3246733 | 81554 | 2.51% |

**HLA-B*40:01**

| Region | Number of people typed (sample size) | Allele count | Allele frequency |
| --- | --- | --- | --- |
| South-East Asia | 22721 | 2914 | 12.83% |
| Australia | 500 | 41 | 8.20% |
| Oceania | 869 | 67 | 7.71% |
| North-East Asia | 26121 | 1342 | 5.14% |
| North America | 2883059 | 116452 | 4.04% |
| Europe | 108656 | 4369 | 4.02% |
| South Asia | 4568 | 80 | 1.75% |
| Western Asia | 195765 | 2702 | 1.38% |
| South and Central America | 3307 | 28 | 0.85% |
| North Africa | 595 | 3 | 0.50% |
| Sub-Saharan Africa | 2150 | 7 | 0.33% |
| Worldwide Total | 3248311 | 128005 | 3.94% |

**HLA-B*46:01**

| Region | Number of people typed (sample size) | Allele count | Allele frequency |
| --- | --- | --- | --- |
| South-East Asia | 22721 | 2654 | 11.68% |
| North-East Asia | 26121 | 1179 | 4.51% |
| North America | 2883059 | 25463 | 0.88% |
| Europe | 108656 | 175 | 0.16% |
| Oceania | 869 | 1 | 0.12% |
| Western Asia | 195765 | 28 | 0.01% |
| Australia | 500 | 0 | 0.00% |
| North Africa | 595 | 0 | 0.00% |
| South and Central America | 3307 | 0 | 0.00% |
| South Asia | 4568 | 0 | 0.00% |
| Sub-Saharan Africa | 2150 | 0 | 0.00% |
| Worldwide Total | 3248311 | 29500 | 0.91% |

**HLA-B*13:02**

| Region | Number of people typed (sample size) | Allele count | Allele frequency |
| --- | --- | --- | --- |
| Western Asia | 195765 | 8750 | 4.47% |
| Europe | 108656 | 4118 | 3.79% |
| South-East Asia | 22721 | 687 | 3.02% |
| North America | 2883059 | 53305 | 1.85% |
| South Asia | 4568 | 64 | 1.40% |
| Sub-Saharan Africa | 2150 | 28 | 1.30% |
| North Africa | 595 | 7 | 1.18% |
| North-East Asia | 26121 | 265 | 1.01% |
| South and Central America | 3307 | 29 | 0.88% |
| Oceania | 869 | 3 | 0.35% |
| Australia | 500 | 1 | 0.20% |
| Worldwide Total | 3248311 | 67257 | 2.07% |

**HLA-C*07:02**

| Region | Number of people typed (sample size) | Allele count | Allele frequency |
| --- | --- | --- | --- |
| South-East Asia | 14802 | 2591 | 17.50% |
| North-East Asia | 22780 | 2903 | 12.74% |
| South and Central America | 2794 | 354 | 12.67% |
| North America | 782952 | 99180 | 12.67% |
| Europe | 106427 | 13134 | 12.34% |
| Oceania | 709 | 84 | 11.85% |
| South Asia | 4376 | 513 | 11.72% |
| Western Asia | 711 | 56 | 7.88% |
| Australia | 355 | 22 | 6.20% |
| Sub-Saharan Africa | 1950 | 97 | 4.97% |
| North Africa | 368 | 16 | 4.35% |
| Worldwide Total | 938224 | 118949 | 12.68% |

**HLA-C*01:02**

| Region | Number of people typed (sample size) | Allele count | Allele frequency |
| --- | --- | --- | --- |
| Australia | 355 | 84 | 23.66% |
| Oceania | 709 | 139 | 19.61% |
| South-East Asia | 14802 | 2494 | 16.85% |
| North-East Asia | 22780 | 3797 | 16.67% |
| South and Central America | 2794 | 283 | 10.13% |
| South Asia | 4376 | 183 | 4.18% |
| North America | 782952 | 32491 | 4.15% |
| Europe | 106427 | 4100 | 3.85% |
| Western Asia | 711 | 12 | 1.69% |
| Sub-Saharan Africa | 1950 | 12 | 0.62% |
| North Africa | 368 | 2 | 0.54% |
| Worldwide Total | 938224 | 43596 | 4.65% |

**HLA-C*06:02**

| Region | Number of people typed (sample size) | Allele count | Allele frequency |
| --- | --- | --- | --- |
| North Africa | 368 | 58 | 15.76% |
| Sub-Saharan Africa | 1950 | 292 | 14.97% |
| South Asia | 4376 | 638 | 14.58% |
| Western Asia | 711 | 94 | 13.22% |
| Europe | 106427 | 10797 | 10.14% |
| North America | 782952 | 66912 | 8.55% |
| South and Central America | 2794 | 134 | 4.80% |
| South-East Asia | 14802 | 459 | 3.10% |
| North-East Asia | 22780 | 357 | 1.57% |
| Australia | 355 | 4 | 1.13% |
| Oceania | 709 | 3 | 0.42% |
| Worldwide Total | 938224 | 79750 | 8.50% |

**HLA-C*03:04**

| Region | Number of people typed (sample size) | Allele count | Allele frequency |
| --- | --- | --- | --- |
| Oceania | 709 | 87 | 12.27% |
| North-East Asia | 22780 | 2745 | 12.05% |
| South-East Asia | 14802 | 1753 | 11.84% |
| South and Central America | 2794 | 232 | 8.30% |
| North America | 782952 | 52779 | 6.74% |
| Europe | 106427 | 6662 | 6.26% |
| Sub-Saharan Africa | 1950 | 97 | 4.97% |
| South Asia | 4376 | 73 | 1.67% |
| Western Asia | 711 | 7 | 0.98% |
| Australia | 355 | 3 | 0.85% |
| North Africa | 368 | 3 | 0.82% |
| Worldwide Total | 938224 | 64442 | 6.87% |

**HLA-C*08:01**

| Region | Number of people typed (sample size) | Allele count | Allele frequency |
| --- | --- | --- | --- |
| South-East Asia | 14802 | 1880 | 12.70% |
| Oceania | 709 | 50 | 7.05% |
| North-East Asia | 22780 | 1598 | 7.01% |
| South Asia | 4376 | 80 | 1.83% |
| North America | 782952 | 11843 | 1.51% |
| South and Central America | 2794 | 33 | 1.18% |
| Sub-Saharan Africa | 1950 | 13 | 0.67% |
| Western Asia | 711 | 4 | 0.56% |
| Australia | 355 | 1 | 0.28% |
| Europe | 106427 | 201 | 0.19% |
| North Africa | 368 | 0 | 0.00% |
| Worldwide Total | 938224 | 15704 | 1.67% |

**HLA-C*03:03**

| Region | Number of people typed (sample size) | Allele count | Allele frequency |
| --- | --- | --- | --- |
| North-East Asia | 22780 | 2831 | 12.43% |
| Oceania | 709 | 59 | 8.32% |
| Australia | 355 | 24 | 6.76% |
| Europe | 106427 | 5071 | 4.76% |
| South-East Asia | 14802 | 615 | 4.15% |
| North America | 782952 | 32218 | 4.11% |
| South and Central America | 2794 | 70 | 2.51% |
| South Asia | 4376 | 87 | 1.99% |
| Western Asia | 711 | 8 | 1.13% |
| Sub-Saharan Africa | 1950 | 18 | 0.92% |
| North Africa | 368 | 2 | 0.54% |
| Worldwide Total | 938224 | 41004 | 4.37% |

**HLA-C*04:01**

| Region | Number of people typed (sample size) | Allele count | Allele frequency |
| --- | --- | --- | --- |
| North-East Asia | 22780 | 2831 | 12.43% |
| Oceania | 709 | 59 | 8.32% |
| Australia | 355 | 24 | 6.76% |
| Europe | 106427 | 5071 | 4.76% |
| South-East Asia | 14802 | 615 | 4.15% |
| North America | 782952 | 32218 | 4.11% |
| South and Central America | 2794 | 70 | 2.51% |
| South Asia | 4376 | 87 | 1.99% |
| Western Asia | 711 | 8 | 1.13% |
| Sub-Saharan Africa | 1950 | 18 | 0.92% |
| North Africa | 368 | 2 | 0.54% |
| Worldwide Total | 938224 | 41004 | 4.37% |
