# Supplementary material for: Rational Design of a Pan-Coronavirus Vaccine Based on Conserved CTL Epitopes
Source: Viruses. 2021 Feb 21;13(2):333. doi: 10.3390/v13020333 (PMC7926959; doi:10.3390/v13020333)
Supplement: Supplementary file 1 [file viruses-13-00333-s001.zip › supplementary DATA/Supplementary Data 3-The mosaic sequence sets for each protein.docx]

**mosaic_S_cocktail**

>seq1

MFLILLISLPTAFAVIGDLNCPLDPRLKGSFNNRDTGPPSISTDTVDVTNGLGTYYVLDRVYLNTTLFLNGYYPTSGSTYRNMALKGTDKLSTLWFKPPFLSDFINGIFAKVKNTKVFKDGVMYSEFPAITIGSTFVNTSYSVVVQPRTINSTQDGVNKLQGLLEVSVCQYNMCEYPHTICHPKLGNHFKELWHLDTGVVSCLYKRNFTYDVNATYLYFHFYQEGGTFYAYFTDTGFVTKFLFNVYLGMALSHYYVMPLTCISRRDIGFTLEYWVTPLTPRQYLLAFNQDGIIFNAVDCMSDFMSEIKCKTQSIAPPTGVYELNGYTVQPIADVYRRKPDLPNCNIEAWLNDKSVPSPLNWERKTFSNCNFNMSSLMSFIQADSFTCNNIDAAKIYGMCFSSITIDKFAIPNRRKVDLQLGNLGYLQSSNYRIDTTATSCQLYYNLPAANVSVSRFNPSTWNKRFGFIEDSVFVPQPTGVFTNHSVVYAQHCFKAPKNFCPCSSCPGKNNGIGTCPAGTNYLTCDNLCTLDPITFKAPDTYKCPQTKSLVGIGEHCSGLAVKSDYCGNNSCTCQPQAFLGWSADSCLQGDKCNIFANFILHDVNNGLTCSTDLQKANTEIELGVCVNYDLYGISGQGIFVEVNATYYNSWQNLLYDSNGNLYGFRDYITNRTFMIHSCYSGRVSAAYHANSSEPALLFRNIKCNYVFNNSLTRQLQPINYSFDSYLGCVVNAYNSTAISVQTCDLTVGSGYCVDYSKNRRSRRAITTGYRFTNFEPFTVNSVNDSLEPVGGLYEIQIPSEFTIGNMEEFIQTSSPKVTIDCAAFVCGDYAACKLQLVEYGSFCDNINAILTEVNELLDTTQLQVANSLMNGVTLSTKLKDGVNFNVDDINFSPVLGCLGSECSKASSRSAIEDLLFDKVKLSDVGFVEAYNNCTGGAEIRDLICVQSYKGIKVLPPLLSENQISGYTLAATSASLFPPWTAAAGVPFYLNVQYRINGLGVTMDVLSQNQKLIANAFNNALHAIQQGFDATNSALVKIQAVVNANAEALNNLLQQLSNRFGAISASLQEILSRLDALEAEAQIDRLINGRLTALNAYVSQQLSDSTLVKFSAAQAMEKVNECVKSQSSRINFCGNGNHIISLVQNAPYGLYFIHFNYVPTKYVTAKVSPGLCIAGNRGIAPKSGYFVNVNNTWMYTGSGYYYPEPITENNVVVMSTCAVNYTKAPYVMLNTSIPNLPDFKEELDQWFKNQTSVAPDLSLDYINVTFLDLQVEMNRLQEAIKVLNHSYINLKDIGTYEYYVKWPWYVWLLICLAGVAMLVLLFFICCCTGCGTSCFKKCGGCCDDYTGYQELVIKTSHDD

>seq2

MFVFLVLLPLVSSQCVNLTTRTQLPPAYTNSFTRGVYYPDKVFRSSVLHSTQDLFLPFFSNVTWFHAIHVSGTNGTKRFDNPVLPFNDGVYFASTEKSNIIRGWIFGTTLDSKTQSLLIVNNATNVVIKVCEFQFCNDPFLGVYYHKNNKSWMESEFRVYSSANNCTFEYVSQPFLMDLEGKQGNFKNLREFVFKNIDGYFKIYSKHTPINLVRDLPQGFSALEPLVDLPIGINITRFQTLLALHRSYLTPGDSSSGWTAGAAAYYVGYLQPRTFLLKYNENGTITDAVDCALDPLSETKCTLKSFTVEKGIYQTSNFRVQPTESIVRFPNITNLCPFGEVFNATRFASVYAWNRKRISNCVADYSVLYNSASFSTFKCYGVSPTKLNDLCFTNVYADSFVIRGDEVRQIAPGQTGKIADYNYKLPDDFTGCVIAWNSNNLDSKVGGNYNYLYRLFRKSNLKPFERDISTEIYQAGSTPCNGVEGFNCYFPLQSYGFQPTNGVGYQPYRVVVLSFELLHAPATVCGPKKSTNLVKNKCVNFNFNGLTGTGVLTESNKKFLPFQQFGRDIADTTDAVRDPQTLEILDITPCSFGGVSVITPGTNTSNQVAVLYQDVNCTEVPVAIHADQLTPTWRVYSTGSNVFQTRAGCLIGAEHVNNSYECDIPIGAGICASYQTQTNSPRRARSVASQSIIAYTMSLGAENSVAYSNNSIAIPTNFTISVTTEILPVSMTKTSVDCTMYICGDSTECSNLLLQYGSFCTQLNRALTGIAVEQDKNTQEVFAQVKQIYKTPPIKDFGGFNFSQILPDPSKPSKRSFIEDLLFNKVTLADAGFIKQYGDCLGDIAARDLICAQKFNGLTVLPPLLTDEMIAQYTSALLAGTITSGWTFGAGAALQIPFAMQMAYRFNGIGVTQNVLYENQKLIANQFNSAIGKIQDSLSSTASALGKLQDVVNQNAQALNTLVKQLSSNFGAISSVLNDILSRLDKVEAEVQIDRLITGRLQSLQTYVTQQLIRAAEIRASANLAATKMSECVLGQSKRVDFCGKGYHLMSFPQSAPHGVVFLHVTYVPAQEKNFTTAPAICHDGKAHFPREGVFVSNGTHWFVTQRNFYEPQIITTDNTFVSGNCDVVIGIVNNTVYDPLQPELDSFKEELDKYFKNHTSPDVDLGDISGINASVVNIQKEIDRLNEVAKNLNESLIDLQELGKYEQYIKWPWYIWLGFIAGLIAIVMVTIMLCCMTSCCSCLKGCCSCGSCCKFDEDDSEPVLKGVKLHYT

>seq3

MKLFLILLVLPLASCFFTCNSNANLSMLQLGVPDNSSTIVTGLLPTHWICANQSTSVYSANGFFYIDVGNHRSAFALHTGYYDVNQYYIYVTNEIGLNASVTLKICKFGINTTFDFLSNSSSSFDCIVNLLFTEQLGAPLGITISGETVRLHLYNVTRTFYVPAAYKLTKLSVKCYFNYSCVFSVVNATVTVNVTTHNGRVVNYTVCDDCNGYTDNIFSVQQDGRIPNGFPFNNWFLLTNGSTLVDGVSRLYQPLRLTCLWPVPGLKSSTGFVYFNATGSDVNCNGYQHNSVADVMRYNLNFSANSVDNLKSGVIVFKTLQYDVLFYCSNSSSGVLDTTIPFGPSSQPYYCFINSTINTTHVSTFVGVLPPTVREIVVARTGQFYINGFKYFDLGFIEAVNFNVTTASATDFWTVAFATFVDVLVNVSATNIQNLLYCDSPFEKLQCEHLQFGLQDGFYSANFLDDNVLPETYVALPIYYQHTDINFTATASFGGSCYVCKPHQVNISLNGNTSVCVRTSHFSIRYIYNRVKSGSPGDSSWHIYLKSGTCPFSFSKLNNFQKFKTICFSTVAVPGSCNFPLEATWHYTSYTIVGALYVTWSEGNSITGVPYPVSGIREFSNLVLNNCTKYNIYDYVGTGIIRSSNQSLAGGITYVSNSGNLLGFKNVSTGNIFIVTPCNQPDQVAVYQQSIIGAMTAVNESRYGLQNLLQLPNFYYVSNGGNNCTTAVMTYSNFGICADGSLIPVRPRNSSDNGISAIITANLSIPSNWTTSVQVEYLQITSTPIVVDCATYVCNGNPRCKNLLKQYTSACKTIEDALRLSAHLETNDVSSMLTFDSNAFSLANVTSFGDYNLSSVLPQRNIHSSRIAGRSALEDLLFSKVVTSGLGTVDVDYKSCTKGLSIADLACAQYYNGIMVLPGVADAERMAMYTGSLIGGMVLGGLTSAAAIPFSLALQARLNYVALQTDVLQENQKILAASFNKAINNIVASFSSVNDAITQTAEAIHTVTIALNKIQDVVNQQGSALNHLTSQLRHNFQAISNSIQAIYDRLDSIQADQQVDRLITGRLAALNAFVSQVLNKYTEVRSSRRLAQQKINECVKSQSNRYGFCGNGTHIFSIVNSAPDGLLFLHTVLLPTDYKNVKAWSGICVDGIYGYVLRQPNLVLYSDNGVFRVTSRVMFQPRLPVLSDFVQIYNCNVTFVNISRVELHTVIPDYVDVNKTLQEFAQNLPKYVKPNFDLTPFNLTYLNLSSELKQLEAKTASLFQTTVELQGLIDQINSTYVDLKLLNRFENYIKWPWWVWLIISVVFVVLLSLLVFCCLSTGCCGCCNCLTSSMRGCCDCGSTKLPYYEFEKVHVQ

>seq4

MIHSVFLLMFLLTPTESYVDVGPDSVKSACIEVDIQQTFFDKTWPRPIDVSKADGIIYPQGRTYSNITITYQGLFPYQGDHGDMYVYSAGHATGTTPQKLFVANYSQDVKQFANGFVVRIGAAANSTGTVIISPSTSATIRKIYPAFMLGSSVGNFSDGKMGRFFNHTLVLLPDGCGTLLRAFYCILEPRSGNHCPAGNSYTSFATYHTPATDCSDGNYNRNASLNSFKEYFNLRNCTFMYTYNITEDEILEWFGITQTAQGVHLFSSRYVDLYGGNMFQFATLPVYDTIKYYSIIPHSIRSIQSDRKAWAAFYVYKLQPLTFLLDFSVDGYIRRAIDCGFNDLSQLHCSYESFDVESGVYSVSSFEAKPSGSVVEQAEGVECDFSPLLSGTPPQVYNFKRLVFTNCNYNLTKLLSLFSVNDFTCSQISPAAIASNCYSSLILDYFSYPLSMKSDLSVSSAGPISQFNYKQSFSNPTCLILATVPHNLTTITKPLKYSYINKCSRLLSDDRTEVPQLVNANQYSPCVSIVPSTVWEDGDYYRKQLSPLEGGGWLVASGSTVAMTEQLQMGFGITVQYGTDTNSVCPKLEFANDTKIASQLGNCVEYSLYGVSGRGVFQNCTAVGVRQQRFVYDAYQNLVGYYSDDGNYYCLRACVSVPVSVIYDKETKTHATLFGSVACEHISSTMSQYSRSTRSMLKRRDSTYGPLQTPVGCVLGLVNSSLFVEDCKLPLGQSLCALPDTPSTLTPRSVRSVPGEMRLASIAFNHPIQVDQLNSSYFKLSIPTNFSFGVTQEYIQTTIQKVTVDCKQYVCNGFQKCEQLLREYGQFCSKINQALHGANLRQDDSVRNLFASVKSSQSSPIIPGFGGDFNLTLLEPVSISTGSRSARSAIEDLLFDKVTIADPGYMQGYDDCMQQGPASARDLICAQYVAGYKVLPPLMDVNMEAAYTSSLLGSIAGVGWTAGLSSFAAIPFAQSIFYRLNGVGITQQVLSENQKLIANKFNQALGAMQTGFTTTNEAFRKVQDAVNNNAQALSKLASELSNTFGAISASIGDIIQRLDVLEQDAQIDRLINGRLTTLNAFVAQQLVRSESAALSAQLAKDKVNECVKAQSKRSGFCGQGTHIVSFVVNAPNGLYFMHVGYYPSNHIEVVSAYGLCDAANPTNCIAPVNGYFIKTNNTRIVDEWSYTGSSFYAPEPITSLNTKYVAPQVTYQNISTNLPPPLLGNSTGIDFQDELDEFFKNVSTSIPNFGSLTQINTTLLDLTYEMLSLQQVVKALNESYIDLKELGNYTYYNKWPWYIWLGFIAGLVALALCVFFILCCTGCGTNCMGKLKCNRCCDRYEEYDLEPHKVHVH

**mosaic_M_cocktail**

>seq1

MSNMTQLTEAQIIAIIKDWNFAWSLIFLLITIVLQYGYPSRSMTVYVFKMFVLWLLWPSSMALSIFSAIYPIDLASQIISGIVAAVSAMMWISYFVQSIRLFMRTGSWWSFNPETNILLNVPLHGTILTRPLLESELVIGAVILRGHLRIAGHHLGRCDIKDLPKEITVATSRTLSYYKLGASQRVAGDSGFAAYSRYRIGNYKLNTDHSSSSDNIALLVQ

>seq2

MSNSSVPLSEVYVHLRNWNFSWNLILTLFIVVLQYGHYKYSRLLYGLKMSVLWCLWPLVLALSIFDCFVNFNVDWVFFGFSILMSIITLCLWVMYFVNSFRLWRRVKTFWAFNPETNAIISLQVYGHNYYLPVMAAPTGVTLTLLSGVLLVDGHKIATRVQVGQLPKYVIVATPSTTIVCDRVGRSVNETSQTGWAFYVRAKHGDFSGVASQEGVLSEREKLLHLI

>seq3

MSSKTTPAPVYIWTADEAIKFLKEWNFSLGIILLFITIILQFGYTSRSMFVYVIKMIILWLMWPITIILTTFNCVYALNNVYLGLSIVFTIVAIIMWIVYFVNSIRLFIRTGSFWSFNPETNCLLNVPIGGTTVVRPLVEDSTSVTAVVTNGHLKMAGMHFGACDYDRLPNEVTVAKPNVLIALKMVKRQSYGTNSGVAIYHRYKAGNYRSPPITADIELALLRA

>seq4

MADSNGTITVEELKKLLEQWNLVIGFLFLTWICLLQFAYANRNRFLYIIKLIFLWLLWPVTLACFVLAAVYRINWITGGIAIAMACLVGLMWLSYFIASFRLFARTRSMWSFNPETNNLMCIDMKGTMYVRPIIEDYHTLTVTIIRGHLYIQGIKLGIGYSLADLPAYMTVAKVTHLCTYKRGFLDRISDTSGFAVYVKSKVGNYRLPSTQKGSGMDTALLRNNI

**mosaic_N_cocktail**

>seq1

MASPAAPRAVSFADNNDITNTNLSRGRGRNPKPRAAPNNTVSWYTGLTQHGKVPLTFPPGQGVPLNANSTPAQNAGYWRRQDRKINTGNGIKQLAPRWYFYYTGTGPEAALPFRAVKDGIVWVHEDGATDAPSTFGTRNPNNDSAIVTQFAPGTKLPKNFHIEGTGGNSQSSSRASSVSRNSSRSSSQGSRSGNSTRGTSPGPSGIGAVGGDLLYLDLLNRLQALESGKVKQSQPKVITKKDAAAAKNKMRHKRTSTKSFNMVQAFGLRGPGDLQGNFGDLQLNKLGTEDPRWPQIAELAPTASAFMGMSQFKLTHQNNDDHGNPVYFLRYSGAIKLDPKNPNYNKWLELLEQNIDAYKTFPKKEKKQKAPKEESTDQMSEPPKEQRVQGSITQRTRTRPSVQPGPMIDVNTD

>seq2

MASVNWADDRAARKKFPPPSFYMPLLVSSDKAPYRVIPRNLVPIGKGNKDEQIGYWNVQERWRMRRGQRVDLPPKVHFYYLGTGPHAKDQYGTDINGVYWVASNQADVNTPADIVDRDPSSDEAIPTRFPPGTVLPQGYYIEGSGRSAPNSRSTSRTSSRASSAGSRSRANSGNRTPTSGVTPDMADQIASLVLAKLGKDATKPQQVTKHTAKEVRQKILNKPRQKRSPNKQCTVQQCFGKRGPNQNFGGGEMLKLGTSDPQFPILAELAPTAGAFFFGSKLELAKVQNLSGNPDEPQKDVYELRYNGAIRFDSTLSGFETIMKVLSENLNAYQQQDGMMNMSPKPQRQRGHKNGQGENDNISVAVPKSRVQQNKSIELTAEDISLLKKMDEPFTEDTSEI

>seq3

MSDNGPQNQRNAPRITFGGPSDSTGSNQNGERSGARSKQRRPQGLPNNTASWFTALTQHGKEDLKFPRGQGVPINTNSSPDDQIGYYRRATRRIRGGDGKMKDLSPRWYFYYLGTGPEAGLPYGANKDGIIWVATEGALNTPKDHIGTRNPANNAAIVLQLPQGTTLPKGFYAEGSRGGSQASSRSSSRSRNSSRNSTPGSSRGTSPARMAGNGGDAALALLLLDRLNQLESKMSGKGQQQQGQTVTKKSAAEASKKPRQKRTATKAYNVTQAFGRRGPEQTQGNFGDQELIRQGTDYKHWPQIAQFAPSASAFFGMSRIGMEVTPSGTWLTYTGAIKLDDKDPNFKDQVILLNKHIDAYKTFPPTEPKKDKKKKADETQALPQRQKKQQTVTLLPAADLDDFSKQLQQSMSSADSTQA

>seq4

MSFTPGKQSSSRASSGNRSGNGILKWADQSDQFRNVQTRGRRAQPKQTATSQQPSGGNVVPYYSWFSGITQFQKGKEFEFAEGQGVPIAPGVPATEAKGYWYRHNRRSFKTADGNQRQLLPRWYFYYLGTGPHKDLKFRQRSDGVVWVAKEGAKTVNTSLGNRKRNQKPLEPKFSIALPPELSVVEFEDRSNNSSRASSRSSTRNNSRDSSRSTSRQQSRTRSDSNQSSSDLVAAVTLALKNLGFDNQSKSPSSSGTSTPKKPNKPLSQPRADKPSQLKKPRWKRVPTREENVIQCFGPRDFNHNMGDSDLVQNGVDAKGFPQLAELIPNQAALFFDSEVSTDEVGDNVQITYTYKMLVAKDNKNLPKFIEQISAFTKPSSIKEMQSQSSHVVQNTVLNASIPESKPLADDDSAIIEIVNEVLH

**mosaic_E_cocktail**

>seq1

MFMADAYLADTVWYVGQIIFIVAICLLVTIVVVAFLATFKLCIQLCGMCNTLVLSPSIYVFNRGRQFYEFYNDIKPPVLDVDDVIQTL

>seq2

MLPFVQERIGLFIVNFFIFTVVCAITLLVCMAFLTATRLCVQCMTGFNTLLVQPALYLYNTGRSVYVKFQDSKPPLPPDEWV

>seq3

MFLRLIDDNGIVLNSILWLLVMIFFFVLAMTFIKLIQLCFTCHYFFSRTLYQPVYKIFLAYQDYMQIAPVPAEVLNV

>seq4

MYSFVSEETGTLIVNSVLLFLAFVVFLLVTLAILTALRLCAYCCNIVNVSLVKPSFYVYSRVKNLNSSRVPDLLV
